# Academic career in medicine – requirements and conditions for successful advancement in Switzerland

**DOI:** 10.1186/1472-6963-9-70

**Published:** 2009-04-29

**Authors:** Barbara Buddeberg-Fischer, Martina Stamm, Claus Buddeberg

**Affiliations:** 1 Department of Psychosocial Medicine, Zurich University Hospital, Zurich, Switzerland

## Abstract

**Background:**

Within the framework of a prospective cohort study of Swiss medical school graduates a sample of young physicians aspiring to an academic career were surveyed on their career support and barriers experienced up to their sixth year of postgraduate training.

**Methods:**

Thirty-one junior academics took part in semi-structured telephone interviews in 2007. The interview guideline focused on career paths to date, career support and barriers experienced, and recommendations for junior and senior academics. The qualitatively assessed data were evaluated according to Mayring's content analysis. Furthermore, quantitatively gained data from the total cohort sample on person- and career-related characteristics were analyzed in regard to differences between the junior academics and cohort doctors who aspire to another career in medicine.

**Results:**

Junior academics differ in terms of instrumentality as a person-related factor, and in terms of intrinsic career motivation and mentoring as career-related factors from cohort doctors who follow other career paths in medicine; they also show higher scores in the Career-Success Scale. *Four types of career path *could be identified in junior academics: (1) focus on basic sciences, (2) strong focus on research (PhD programs) followed by clinical training, (3) one to two years in research followed by clinical training, (4) clinical training and research in parallel. The interview material revealed the following *categories of career-supporting experience*: making oneself out as a proactive junior physician, research resources provided by superior staff, and social network; statements concerning career *barriers *encompassed interference between clinical training and research activities, insufficient research coaching, and personality related barriers. *Recommendations for junior academics *focused on mentoring and professional networking, *for senior academics *on interest in human resource development and being role models.

**Conclusion:**

The conditions for an academic career in medicine in Switzerland appear to be difficult especially for those physicians combining research with clinical work. For a successful academic career it seems crucial to start with research activities right after graduation, and take up clinical training later in the career. Furthermore, special mentoring programs for junior academics should be implemented at all medical schools to give trainees more goal-oriented guidance in their career.

## Background

Depending on the career aspired to, medical school graduates plan their postgraduate training differently. In Switzerland, as in other German-speaking countries, nearly half of all young physicians aspire to work as a medical specialist in a private practice [1]. Another forty per cent pursue a hospital career, but only about ten percent aspire to an academic career. As found in the SwissMedCareer study [2,3] and reported by other authors [4-6], *women physicians are generally less interested in academic pursuits than men*. Along with the feminization of medicine new challenges arise, if women are not as much interested in an academic career or if they experience career obstacles so that they withdraw from this career plan, as a consequence there might be a lack of young academics in the near future. At the beginning of specialty training a greater number of residents think of an academic career but lose interest in it later on [7]. The main reasons assessed are lack of mentorship and role models, absence of faculty development programs, financial issues and difficulties balancing work and family [8,9]. Independently of the career aspired to, *performance should be assessed at regular intervals *to provide trainees with adequate career support [2,10]. It has been recognized that faculties need formalized career development strategies to improve the progress of their trainees [11,12]. *Mentorship *has been identified as an important component of personal development, career guidance, choice, and success [13,14]. Experience in a mentoring program [15] has shown that, especially with women, continuous follow-up of young physicians' career advancement and feed-back on their performance motivates them not to abandon their initial career aspiration. Higgins et al. [16] and Blickle et al. [17] postulated that not only the quality of a mentor-protégé relationship but also the sum of different supporting relationships, forming a kind of supporting network, is career relevant. Beside institutional career support, a career-oriented *professional attitude and personality traits *such as instrumentality (i.e. to be decisive, independent, proactive and goal-oriented) are positively associated with objective indicators of career success [18,19]. Furthermore, the *personal situation *often has an influence on career aspirations and success, in the sense of parents being role models (being academics), of receiving support from a partner, and of the amount of domestic commitments; the latter two factors being more important for women [20].

In Switzerland, as in the other German speaking countries, physicians who aspire to a career in academia have to be successful researchers, regardless of their clinical career. In Switzerland, the first step on the hierarchical ladder is called 'habilitation'. The requirements for a habilitation qualification are at least 15 – 20 papers published as original papers in peer-reviewed English language journals with a high impact factor in the field, being first author in at least half of the papers. Furthermore, the applicants need to have acquired competitive research grants. The third requirement is experience as a lecturer. The next career step is called 'Titularprofessor', for which another 12 original papers are required. 'Titularprofessors' are not faculty members. The next steps are associate professor and full professor. The number of these posts at university hospitals and/or medical schools is limited in Switzerland. A vacant post has to be internationally announced. A special expert commission is established to thoroughly assess the applicants professionally and personally, and finally to decide who is to be elected. The election procedure is very competitive. However, networking plays an important role. The old boys' network of male professors discriminates against young female academics. Unfortunately there are not alternative academic career paths in Switzerland such as clinician-educators in the US [21].

Within the framework of a prospective survey of a cohort of Swiss medical school graduates [22-24], it was investigated first how many of the cohort aspire to an academic career at the end of their residency, and at what time of their postgraduate training they came to this decision; second in what characteristics those aspiring to an academic career differ from those aspiring to other career paths in medicine in terms of person- and career-related factors, based on quantitatively collected data. As mentioned, the interest in an academic career is declining. There is a need for further information about what has to change in postgraduate training to increase the young physicians' interest in an academic career. Therefore, semi-structured telephone-interviews with junior academics (participants in the SwissMedCareer study) were conducted to investigate (1) what career paths the junior academics have followed, (2) what kind of career support they received, (3) what career barriers they experienced, (4) what career recommendations they give other junior staff, and (5) what they recommend senior staff should do to improve career support. The present paper mainly focuses on the qualitative data.

## Methods

### Study design, sample development and study sample

The present study is part of an ongoing *prospective survey of a cohort of graduates *of the three medical schools in German speaking Switzerland [22-24], beginning in 2001 (T1). Subjects were re-evaluated every two years. At the fourth assessment (T4) in 2007 the participants had worked as residents for five to six years; i.e. they had the same seniority at this stage of their career, similar positions and salary. The participants were asked what kind of career they aspired to (private practice, hospital medicine, academic medicine, other medical fields). Of the total sample at T4 (n = 404), 41 aspired to an academic career: 9 (22%) were females and 32 (78%) males, mean age was 32.2 years (SD 1.3 yrs, range 29 – 37 yrs), 8 (89%) females and 28 (88%) males lived with a partner, children had none of the females, but 4 (13%) of the males. The other 363 study participants aspired to a hospital career, to work in a private practice or in another medical field: of these 197 (54%) were females and166 (46%) males, mean age was 33.3 years (SD 2.1 yrs, range 30 – 47 yrs), 161 (82%) females and 144 (87%) males lived with a partner, children had 34 (17%) of the females and 46 (28%) of the males.

Chosen specialty in those study participants aspiring to an academic career and those aspiring to another career in medicine is shown in Table 1. In female academics surgery, pediatrics, and other specialties (dermatology, neurology, nuclear medicine, pathology) as chosen specialty is overrepresented and internal medicine underrepresented compared to female physicians choosing other career paths. In male academics also surgery and pediatrics are chosen more often, internal medicine and anesthesiology less often.

**Table 1:** Specialty aspired to in junior academics (n = 41) and in participants aspiring to another career in medicine (n = 363) according to gender

	**Academic career**		**Other career in medicine**	
	**Females** n (%)	**Males** n (%)	**Females** n (%)	**Males** n (%)
**Specialty aspired to**				
- Primary Care	0	0	29 (14.7)	20 (12.0)
- Internal Medicine	1 (11.1)	6 (18.8)	56 (28.5)	48 (29.0)
- Surgery	3 (33.4)	12 (37.5)	11 (5.6)	30 (18.1)
- Gynecology	0	0	25 (12.7)	1 (0.6)
- Anesthesiology	1 (11.1)	1 (3.1)	18 (9.1)	16 (9.6)
- Pediatrics	2 (22.2)	4 (12.5)	23 (11.7)	5 (3.0)
- Psychiatry	0	0	15 (7.6)	10 (6.0)
- Other specialty	2 (22.2)	5 (15.6)	15 (7.6)	27 (16.3)
- no clinical field	0	4 (12.5)	5 (2.5)	9 (5.4)

Total	9 (100.0)	32 (100.0)	197 (100.0)	166 (100.0)

No computation of significance possible because of too small cell sizes.

To investigate the issues of the present paper, those participants aspiring to an academic career (n = 41) were approached. Thirty-one took part in a telephone interview, of the others one subject refused to participate, nine subjects did not answer. The interview material of 31 subjects (6 females, 25 males) could thus be included in the content analysis. *The respondents and the non-respondents did not differ in terms of personal and career-related characteristics *(Wilk's Lambda = 0.75, F(7,32) = 1.56, p = 0.182).

The returned questionnaires were only identified by a code in order to ensure participant anonymity. The respondents sent their addresses to an independent address-administration office, allowing for follow-up. The study protocol was approved by the Ethical Committee of Zurich University.

### Instruments

#### Quantitative data

All instruments are self-assessment scales. The main characteristics of the instruments and the constructs measured by them are described in the following:

- *Questions concerning socio-demographic data*

- *Career aspired to *(private practice, hospital or academic career), and *chosen medical specialty*

- *Sense of Coherence Scale (SOC-13)*[25] (seven-point Likert scale), a measure of a person's resistance to stress and his/her ability to manage stress.

- *Personal Attributes Questionnaire*, *GE-PAQ*, German Extended Personal Attributes Questionnaire [26], a self-rating instrument for the assessment of gender-role orientation. Only the *Instrumentality (PAQ-I) *scale (8 items, six-point Likert scale) was used which contains instrumental traits (e.g. independent, decisive, to be proactive, goal-oriented) considered socially desirable to some degree in both sexes but stereotypically more characteristic of males.

- *Career-Motivation Questionnaire *[27] (seven-point rating scale), *Intrinsic *(i.e. enjoyment of and interest in professional activities) and *Extrinsic Career Motivation Scale *(i.e. striving for promotion, income, prestige) and the *Extraprofessional Concerns Scale *(i.e. prioritizing family, convenient working hours, job security) were applied.

- *Mentor-Protégé Relationships Questionnaire *[17] consisting of five scales (Likert scale 0 – 4) measuring career-support type. We only used the *Networking scale *(4 items) and the *Support in career planning *scale (3 items). These two scales describe the crucial aspects of mentoring. Our data analyses show that the two scales are highly correlated (r = 0.71). We therefore combined them into one scale named "*Mentoring-Experience Scale (MES)*", Cronbach's alpha = 0.92.

- *Career-Success Scale (CSS) *[2] consisting of 7 items addressing scientific activities (number of research projects, lectures, publications, grants, criteria which correspond to the requirements for tenure track)

#### Qualitative data

The telephone interviews conducted by the second author were structured by an interview guideline. It focused on five topics (domains): (1) career path to date, (2) career support received, (3) career barriers experienced, (4) recommendations for junior academics and (5) for superiors.

### Statistical analyses

All quantitative analyses were carried out with SPSS for Windows, release 15.0. Descriptive statistics are given in terms of counts and percentages, means and standard deviations respectively. Differences between groups of physicians were tested with multivariate analyses of covariance (covariate: gender) naming Wilk's Lambda and Bonferroni post-hoc tests.

The qualitatively assessed data (interview material) were evaluated according to Mayring's [28] content analysis. The telephone interviews were tape-recorded and transcribed. The assignment of statements to the five domains of the interview guideline was made independently by the authors in a first step and adapted after joint discussion in a second step. In the following, the material of each domain was assigned to inductively gained categories. This procedure was discussed twice in a workshop for researchers at the department of Psychosocial Medicine and completed by the first two authors.

## Results

### Academics and non-academics: person- and career-related factors

At T4, six years after graduation, 41 cohort doctors aspired to an academic career. At T2, in their second year of residency, only 16 of them planned this career path, and at T3, in their fourth year of residency, 24 of them followed this goal. Table 2 lists the person- and career-related characteristics of the cohort doctors aspiring to an academic career (n = 41) and those aspiring to another career in medicine (n = 363). Junior academics, irrespectively of gender, rated themselves as being more instrumental (i.e. decisive, proactive and goal-oriented), and as having higher intrinsic career motivation, lower extraprofessional concerns and as receiving more mentoring. As expected, junior academics showed higher scores in the Career Success Scale. As mentioned, the interview respondents (n = 31) and non-respondents (n = 41) did not differ in terms of personal and career-related characteristics.

**Table 2:** Means and standard deviations (SD) of person- and career-related factors in junior academics (n = 41) and in participants aspiring to another career in medicine (n = 363); results of multivariate analysis of covariance with gender as covariate

	**Academic career ** **mean (SD)**	**Other career in medicine ** **mean (SD)**	**univariate p**	**univariate partial eta^2^**
Sense of Coherence SOC-13	5.07 (0.85)	5.10 (0.86)	0.640	0.001
PAQ Instrumentality	4.51 (0.49)	4.19 (0.68)	0.026	0.012
Intrinsic career motivation	6.33 (0.42)	6.13 (0.50)	0.030	0.012
Extrinsic career motivation	4.02 (0.71)	3.69 (0.85)	0.188	0.004
Extraprofessional concerns	3.80 (1.06)	4.33 (1.00)	0.004	0.020
Mentoring	2.59 (0.94)	1.69 (0.98)	< 0.001	0.047
Career Success Scale	6.24 (2.80)	1.28 (1.65)	< 0.001	0.387

Wilk's Lambda = 0.60, F(7,395) = 37.45, p < 0.001, partial eta^2 ^= 0.399

### Career paths of junior academics interviewed

Of the junior academics interviewed (n = 31) there were 25 males and 6 females, 29 lived with a partner, 4 males had children, and none of the females. In terms of chosen specialty, 11 (35%) had chosen surgical fields, 6 (19%) pediatrics, 5 (16%) internal medicine, 2 (7%) anesthesiology, and 5 (16%) other specialties (2 dermatology, 1 neurology, 1 nuclear medicine, 1 pathology), one each (6%) specialized in epidemiology and biomedical statistics (non-clinical subjects).

Among the 31 interviewees *four types of career path *can be distinguished. *The first type is a career path focused on research activities*. This path was chosen by four male participants, three having attained a PhD degree and 1 having completed postgraduate studies in statistics. *The second type of career path included a strong focus on research or further education and a subsequent focus on clinical training with the aim of attaining a medical specialist title*. This second type of career path was typical of 5 male participants, 4 having completed a PhD degree and 1 having completed a degree in dental medicine, all aspiring to a medical specialist title at the time of the interview. *The third type of career path comprised a year (at least) of research activities alternating with clinical training, with the aim of attaining a medical specialist title*. This pattern could be found in 8 participants (2 females, 6 males). The remaining 14 participants (4 females, 10 males) showed a career path *(fourth type) which combined clinical training and research at the same time*. Often the research activities were practiced in addition to full time clinical activity. Some participants focused on research for a few months and some were allocated a certain number of hours for research within their clinical employment.

### Process of receiving career support

In the interviews the respondents described the various facets of career support they received to date, how they experienced advancement, and which determinants turned out to be essential for a successful career path. Table 3 lists the main answers for each of the five categories of career support deduced from the interview material.

**Table 3:** Career support received

**Category**		**Examples**	
1.	Making onself out as a proactive junior academic	1.	I had to make an active effort to obtain support. The research project came about because we discussed it and I volunteered to tackle it.
		2.	I did not know my superior personally and tried to join his research group. As we were both happy to work together, things developed from there.
		3.	Projects were worked out together. The senior physician gave me ideas and supported me in putting them into practice.

2.	Motivating activities by senior academics and mentors	4.	My mentor herself was very actively involved in mentoring. She had spent 3 years in America where they have mentoring programs.
		5.	My thesis supervisor approached me, we worked his ideas out together, I then put most of them into practice on my own. In time I also developed my own projects and presented them to the relevant people. It was a fluid transition towards independence.
		6.	I was in a mentoring program for 1 year. On the whole it was helpful. Everyone was allocated a mentor. I had a professor of anesthesiology. He tended to give me general advice on career issues. As he was in a different specialty he could not provide much help in building up a network. In the mentoring program we also had a few workshops on the qualifications you needed to be eligible for habilitation, how to plan this route and how you could continue academic work in parallel to specialist clinical training.
		7.	In the children's hospital in Bern we have a mentoring project which I joined. My mentor worked in the same hospital and had some influence there. Thanks to this person we were able to get some research projects and my further clinical training back on track.

3.	Supportive senior staff at different hierarchical levels	8.	In Basel the medical school course includes primary care physician training. And this gave rise to a relationship with a family doctor in Basel who now advises me. He has become a mentor to me, especially on the personal level. I also receive a lot of encouragement from the Professor. This is a friendship which arose from my being able to solve his computer problems. I pose no danger to a professor about to retire (although I have no intention of being a danger). I am also supported by colleagues who are a year ahead of me; we are all in this together, everyone has the same problems.
		9.	I received support from senior physicians and heads of department, more in the sense of informal mentoring. Part of the support I received was to ease the pressure in hospital, especially when we wanted to carry out joint clinical studies.
		10.	I receive support from my current superior in the sense of mentoring and career planning, also from the laboratory research director, a natural scientist who helps me in my research.

4.	Research resources provides (time, infrastructure, money)	11.	I received most support from my thesis supervisor, he made it possible for me to spend a year in academia and also financed this with third party funds, he also taught me the academic skills.
		12.	In Bern I had someone who supported me in carrying out research and gave me free time to do it. I was also given access to clinical data and software.
		13.	My superior suggested that I could now spend 80% of my time on clinical work and continue doing research for 20%.
		14.	I was given support both in planning basic research and in implementing projects, also in how to draw up project applications, present research results to conferences and describe them in publications.
		15.	All the conferences I attend are fully paid for, including hotel, flight and car hire. An additional qualification, Master of Science and Clinical Research, is now being funded (CHF 25,000). I am also given the time for this.

5.	Social network and family	16.	My husband supports me in every way he can. A woman cannot forge a career without a tolerant partner.
		17.	My partner is a biologist and works in research, I discuss quite a lot with her, including future plans, e.g. going abroad.
		18.	In my private life there is my wife who is a clinician; she naturally knows what my job entails, can understand my problems and helps me move forward through discussion.
		19.	My father-in-law (unfortunately dead now) was also active in research, I had lots of career-specific discussions with him.
		20.	My friends support me through our common leisure activities, which balance work.
		21.	I received some financial support from my family and parents.

#### 1. Making oneself out as a proactive junior physician

The first question posed was how the junior academics experienced the process of receiving career support up to date. A similar process was described by most of the respondents: They actively approached superiors, asked them for projects to collaborate on, and for support in planning their own studies. Being proactive and acting on one's own initiative proved to be crucial attitudes and behavior by which ambitious and smart junior staff members were recognized by senior academics. If junior staff turned out to be committed, senior staff approached them for cooperation in research projects. Over time a reciprocal relationship between junior and senior staff was established in most cases. Senior staff supported the junior staff with project ideas, helped them to establish a research network, funded their studies with grants, or allowed them protected research time (see statements 1 – 3).

#### 2. Motivating activities by senior academics and mentors

Some senior academics acted on their own initiative and approached junior staff members to be their coach or mentor. This was the case with senior academics who had a mentorship experience in their own career, mainly in US medical faculties (statement 4). Some respondents reported that their doctoral thesis adviser turned out to be their first mentor (statement 5): if the professional and personal level fitted well, the relationship turned out to be supportive in the long run. In some Swiss medical faculties, mentoring programs have been established. Four of the subjects interviewed participated in formal mentoring programs in which mentor – mentee matching was assigned by the program managers. If the mentor was a representative from a medical discipline other than that of the mentee, counseling focused mainly on general career advise (statement 6). If mentor and mentee came from the same discipline, career support was more specific and helped to establish a professional network (statement 7).

Summarizing the statements on the first two issues, it can be stated that *career support is a dynamic interaction process between a proactive junior and a senior academic interested in junior staff members. Reciprocity in regard to commitment and mutual reward between the two partners is essential to guarantee a supportive relationship over time*. It should be borne in mind, however, that a young physician must emanate a certain "charisma" of interest and commitment, otherwise this process does not get off the ground.

#### 3. Supportive superior staff at different hierarchical levels

The respondents specified those who had mainly supported them (statements 8 – 10). Two thirds of the supportive senior staff were heads of department, the others held the position of senior physicians, some were still residents; peer support comprised companionship within a group of junior researchers struggling with similar problems.

#### 4. Research resources provided (time, infrastructure, money)

Personal advice and coaching are important, but it is essential for an academic career to get the relevant resources for research. Resources comprise time for planning projects, writing grants and papers, money to carry out studies and attending conferences. For those junior staff who specialize in a clinical discipline it is especially difficult to combine research activities with clinical work. They therefore have to get protected research time or a one-year research post during their residency (statements 11 – 15).

#### 5. Social network and family

Not only professional support but also a private social network is essential to cope with the challenges of an academic career. The answers of the academics interviewed indicated that spouses play an important role in emotional and intellectual support. A spouse who has an understanding of the amount of time the academic partner is spending on his/her work, who is willing to let his/her own wishes take second place or to take over most of the chores contributes a lot to the well-being and successful career advancement of the academic spouse (statements 16 – 21).

### Experienced career barriers

Another main topic on which the junior academics were interviewed was the issue of career barriers experienced. This area produced eight categories (see Table 4).

**Table 4:** Career barriers

**Category**		**Examples**	
1.	Lack of structured residency programs	1.	Job planning for further training is not very transparent in many specialist fields and hospitals. It is a nuisance having to apply for a job 2 years in advance. Often you do not even get an offer for the whole period of further training. There are still too few further training associations for individual specialist fields.
		2.	There are hardly any structured further training programs for physicians with academic and clinical interests.
		3.	In further training you have to be very flexible about where you live and work. I tend to be the social type who wants to stay near family and friends. I would certainly have progressed faster if I didn't mind where I worked.

2.	Interference between clinical training and research activities	4.	It is difficult to carry on further clinical training and research in parallel. You need to work at least 50% of the time for specialist training, otherwise it is not recognized. There are very few jobs split between 50% research and 50% further clinical training. It is easier to split a job after becoming a specialist.
		5.	It is tremendously difficult to be good at research AND in hospital. I don't know whether I will manage it. It is a great strain, you have to find a way with which you yourself are happy. Clearly a hospital provides services and so you have to be a clinician first and foremost. If I were asked which was more important, a patient or a research project, there would be no question about it being the patient.
		6.	If you aspire to an academic career you still have to do the clinical work. You are relied upon to work in your free time.

3.	Insufficient research coaching	7.	It is difficult to find anyone to initiate you into project planning and research methodology. You always have to keep asking.
		8.	It is not easy to find a research group with a good atmosphere where you are respected but where you also know what you have to achieve, what the targets are.
		9.	Far too little support is given in working out projects for a Swiss National Science Foundation grant and for all the preparation and administrative aspects of a stay abroad.
		10.	Whether a paper is accepted for a journal depends not only on scientific quality, a part is often played by connections and inside contacts.

4.	Demotivating rivalry	11.	I have been thwarted by superiors on many occasions. If you are young and successful and achieve recognition from outside you are sometimes perceived as a potential competitor by your superiors. Consequently they do not let you operate. But you are absolutely dependent on this as a surgeon, without operations you cannot become a surgeon; this knocks your motivation.
		12.	Although a lot of senior staff assert that they give us junior academics long-term support, they are often more interested in being listed as co-authors in publications even though they have had hardly any involvement in the projects. But you can't have it out with people because later on you might want to do something in the research line and need a job. As soon as you scare someone off you can say goodbye to further clinical training.

5.	Financial short-comings	13.	I earn very little in research (CHF 3,500 per month). At 32 it is not pleasant to have to rely on financial support from your parents. It is off-putting when, with all the skills you have acquired to date (technical, intellectual etc.), you have to start at the bottom again. My colleagues who are medics are generally already in senior physician posts and colleagues who work in industry all earn a great deal more than I do.

6.	Personality related barriers	14.	Tolerance to frustration and staying power are very important factors in the academic field. Sometimes you can't make any headway for 6 months but still have to motivate yourself to carry on; then you generally make a breakthrough some time afterwards.
		15.	Your own doubt can be the worst hurdle. You begin to wonder whether you are on the right track, whether you are capable enough or good enough.
		16.	There are difficulties and hurdles everywhere. I see the whole thing as a sporting challenge.

7.	Gender related barriers	17.	Women approach things differently, they are less self-assertive and aggressive than men, are more hesitant about aspiring to an academic career at all, and wonder whether it is worth all the effort.
		18.	Not many senior staff believe that women can make a career in surgery. It is hard work convincing them.
		19.	It never occurs to senior staff that a woman could habilitate, this happens a lot faster for a young, ambitious man.
		20.	The most important thing is self-confidence. You are always hearing that a woman cannot have an academic career and a family at the same time.

8.	Work-family Imbalance	21.	It is quite difficult to introduce part-time working to many specialist fields, for example intensive-care units. It is often thwarted by management. This is a great career obstacle for assistants who have children and aspire to a challenging career.
		22.	Balancing career planning and family planning is a big problem. It also depends on the partner's attitude. Many women tend to put their career plans on hold to give themselves more time to attend to family chores and leave the man free to pursue his career. If you are a woman aspiring to an academic career you will not start a family until relatively late in life, so you have to expect to be an "old" mother and be fairly certain of not just having a half-time job.

#### 1. Lack of structured residency programs

The first category encompassed difficulties in organizing residency training (statements 1 – 3). In several medical specialties the residency training is not properly structured; trainees have to apply for a residency post at least two years before graduation, sometimes they do not even have the opportunity to get to know the characteristics of a specialty; there is no residency matching program; quite often who gets a post and who does not is down to chance; trainees have to change their training institution during residency, which means they have to move to other locations; this is quite often difficult for double career partnerships.

#### 2. Interference between clinical training and research activities

An issue mentioned by most of the respondents was the problem of combining clinical training with research activities (statements 4 – 6). Several barriers were mentioned: there are only a few residency posts which allow research and clinical training to be carried out in parallel. Graduate trainees preferably start their scientific career working in research full-time for the first one to two postgraduate years; they gain a quicker and more fundamental research basis. But when they enter clinical training they often have no protected research time and not enough resources to keep up with their research projects. There are hardly any residency posts where the clinical skills of young academics are promoted and their research work is supported.

#### 3. Insufficient research coaching

Most of the trainees go in for research only after some years of clinical training. They have to ask research group leaders to be involved in some of the studies. It depends entirely on the coaching skills, willingness and fairness of the senior researchers as to whether a junior researcher is well instructed in planning and conducting studies and successfully writing and publishing articles. Furthermore, senior researchers should introduce junior staff to their scientific network so that they can get their studies published in high-ranked journals (statements 7 – 10).

#### 4. Demotivating rivalry

Some interviewees stated that although most chief physicians claim that promoting junior researchers is their special task, there are some heads who get anxious when junior academics are too successful. Sometimes they start to thwart the junior staff in their further career. Others do not really support the junior staff in their research but only make sure that they are on the authors' list of published papers. Even if the head has contributed nothing to the conduct of a research project he claims to be senior author (statements 11 – 12).

#### 5. Financial shortcomings

Most research posts are only paid as a 50% employment job with 100% workload or even more. For those junior staff who are already advanced in their clinical specialty and have passed their specialty qualification exam, it is not easy to accept being salaried like an intern again. Especially those academics who already have a family are dependant on being financially supported by parents or spouses. This often makes them feel uncomfortable (statement 13).

#### 6. Personality related barriers

Another issue addressed the personality related barriers (statements 14 – 16). The respondents were self-critical: a junior researcher has to put up with frustration and failure, has to show stamina, otherwise he/she will not succeed; they have to be self-confident; if they hesitate too much they will not advance on their career path.

#### 7. Gender related barriers

In academia women are still under-represented, so the interviewer especially asked the junior staff about gender related career barriers (statements 17 – 20): Females often doubt whether they have the strength to struggle with the time-consuming and highly demanding requirements of an academic career. They hesitate as to whether the great effort is worth the reward they will get. Often they do not dare to ask superiors for cooperation in a research group, they wait to be asked, and this does not happen as naturally as with male junior staff. In addition to these internal barriers women academics often experience external obstacles: they have to overcome the gender stereotypes still in the minds of chief physicians.

#### 8. Work-Family Imbalance

Some of the gender related barriers are associated with difficulties in balancing career requirements with the needs of a family (statements 21 – 22). Again there are women's internal barriers: mostly they are more willing to put their career on hold to look after the children. On the other side there are institutional obstacles: to date there are not enough part-time jobs for postgraduate training; furthermore, senior staff do not respect gender specific career models which would enable female junior staff to follow their career despite having children.

*The respondents' statements on the process of getting career support and experiencing career barriers can be summarized as follows*: Those junior academics who successfully advance in their career are proactive, well motivated and coached by superiors, receive sufficient research resources, and have a good social network; they are able to accumulate career-supporting factors and overcome career barriers.

### Recommendations for junior academics

The aim of the fourth topic addressed in the interview was to draw up recommendations to help junior academics in achieving successful career advancement (see Table 5).

**Table 5:** Recommendations for junior academics

**Category**		**Examples**	
1.	Career planning	1.	You should consider the career path you want to take at an early stage, pursue it single-mindedly, think ahead, plan forward, think about what an academic career means, be clear about the amount of time involved, plan a stay abroad in good time.
		2.	You should start on research right after finals or perhaps even complete a PhD course and only then continue clinical training. If you take on a clinical job immediately and want to establish yourself academically at the same time, the effort involved makes this extremely difficult. They are both so different from a methodological point of view that it is hard to cope with them together. If you can concentrate on academic work for a while you learn working techniques, are able to go into things in more depth and set a few projects in motion. This makes it much easier to find your feet.
		3.	I would recommend that those who want to make a career in surgery go straight into a large hospital.
		4.	Early on you should join an established research team with a good team culture. In such a group you should actively approach people who can advise you and provide a good introduction to academic work.

2.	Interest in and enjoyment of research	5.	You have to be deeply interested in research and must not stop enjoying it. Quality of life is better if your work gives you pleasure.
		6.	You need power, frustration tolerance and stamina (like a marathon runner or mountaineer, not like someone in the pub).

3.	Mentoring/Networking	7.	The most important thing of all is to have mentors, firstly as personal advisors and secondly as scientific role models. You should actively seek mentors out. It is difficult to fight your way through alone. At the start it could even be a peer mentor who is a bit ahead of you, but later on an advanced academic should definitely take on the mentoring role; sometimes this can be the head of department or another superior.
		8.	It is important to have a large network, including those of similar age who are somewhat further on.
		9.	You have to build a definite network for yourself, not only at local but also at international level. Switzerland is too small to manage without networks. You learn from others the right and wrong moves they have made in their careers.

#### 1. Career planning

Junior academics should plan their intended career at an early stage of their postgraduate training. If they are already sure of the career they want to pursue, it is even better to arrange training posts before graduation. They should preferably start their postgraduate training in a well-known and well organized research group or within a PhD program where they are coached and supervised in basic research activities. Those who specialize in a clinical subject can enter residency training afterwards. If a superior is interested in the research activities of his/her staff he/she will allow the junior members protected research time within the clinical work, enabling junior staff to continue their studies and to write papers (statements 1 – 4).

#### 2. Interest in and enjoyment of research

Becoming an academic is a demanding career path, which involves sacrificing time, money and some private life. Only if a junior academic is enthusiastic about his/her research activities can he/she cope with these challenges. Quality of life is well balanced as long as the work is still enjoyable. Junior academics must learn to cope with failure and overcome feelings of frustration (statement 5 – 6).

#### 3. Mentoring/Networking

Junior academics stated that it is crucial to have a mentor for professional and personal support, to give advice, and to provide a role model. At the beginning of a career peers can also take the role of peer-mentors. Mentors help to establish professional networks which are essential for promotion in the scientific community (statements 7 – 9).

### Recommendations for senior staff

The last interview section dealt with participants' recommendations for the successful support of junior academics by senior staff (see Table 6).

**Table 6:** Recommendations for superiors

**Category**		**Examples**	
1.	Human resource development	1.	Identify colleagues with the qualities for an academic career early on, then actively motivate and support them in such a career.
		2.	From the outset hold regular career talks in which presentations and career opportunities are discussed and subgoals are recorded in writing. Hold follow-up discussions to check whether the career is progressing as planned.
		3.	Provide young academics with structured coaching on how projects are planned and implemented and the best ways to publish research results. This will help them make faster career progress.
		4.	Establish contact with other research groups and send junior staff to conferences so that they can make contacts for themselves.
		5.	Show more appreciation of the hard work and dedication of young academics.
		6.	At head of department level show a willingness to make protected research time available to clinical trainees so that they have enough time to pursue their studies successfully. Encourage junior academics by providing good basic conditions such as time, infrastructure and research funds.

2.	Role model	7.	Senior academics should be role models, radiating enthusiasm for their work and their research. As a role model show how different fields such as clinical activity, research and private life can be managed simultaneously with good organization and good resources. This will give young doctors a hopeful and motivating perspective on their career. Senior staff should provide motivated junior academics with a vision.

#### 1. Human resource development

Senior staff should identify trainees who are ambitious, smart and interested in an academic career at an early stage of their postgraduate training. They should actively motivate and support them in their career. Regular coaching and assessment sessions should be held to define career steps and evaluate career progress. The junior academics should be rewarded for their research effort by protected research time, infrastructure, and funding. It is furthermore essential that superiors push junior staff to present their work at conference and to establish professional networks (statements 1 – 6).

#### 2. Role model

Senior academics can act as role models for junior staff not only by showing enthusiasm in their research activities but also by demonstrating how to manage as a good researcher, an excellent clinician, and still have time for partnership, family and leisure (statement 7).

## Discussion

Within the framework of a prospective Swiss cohort study [3], the present paper reports on qualitative data gained in telephone interviews with study participants aspiring to an academic career. The interview focused on the type of career path, the career support and career barriers they had experienced to date, and the recommendations they can pass on to junior academics and senior staff. Furthermore, the junior academics were compared with those study participants following another career in medicine in terms of quantitatively gained person- and career-related characteristics.

### Characteristics of junior academics

The junior academics were characterized by comparison with those aspiring to another career in medicine. Trainees who pursue an academic career need to be decisive, proactive, goal-oriented, resistant to frustration, and self-confident [2,18,19]. These *person-related characteristics *were also seen more distinctly in our sample of junior academics than in those of the other career paths. As already found in other studies, *mentoring *proved to be an essential determinant of career success [2,10,13,17]. Junior academics experienced more career support in terms of mentoring than the other cohort doctors. The qualitatively gained statements reflected the quantitatively assessed data: The interviewees confirmed the importance of being mentored. As indicated by our data, an important key for career success is the interactive process of career support-seeking by junior academics and career support provision by senior staff.

### Career aspiration and gender

As reported in the literature [4-6,23,29,30], more male physicians aspire to an academic career, and are in the top ranks of faculty posts [31]. Though there is a greater awareness of gender equality to date, gender equity in terms of fairness and justice for men and women in the professional opportunity structure is far from being realized. Whether female physicians themselves follow different professional and personal career goals than males, or whether they do not have equal access to academic career-relevant resources, cannot be distinguished by data of the present study. In the total cohort more than half of the participants are female but only one in five junior academics is a woman. The female physicians interviewed identified several gender related barriers: first of all, women do not mark themselves out as being interested in research and an academic career as distinctly as their male colleagues do; they are less proactive and have lower professional self-efficacy, and are often hesitant as to whether they will be able to balance work and family obligations. Similar findings are reported by other authors [32]. Just as important are obstacles which have their seeds in the gender stereotypes of superiors: Superiors do not believe that women are as interested in and as capable of an academic career as men. Superior staff assume from the first that women trainees will prioritize family concerns over professional ambitiousness [33]. As mentioned in a Norwegian study [34], women need more positive signals on being wanted as researchers.

### Career determinants

Key career determinants reported by the junior academics interviewed related to four domains: (1) decision to follow an academic career already made by the end of medical school; careful planning of postgraduate training; and clear communication to senior staff of the career aspired to. (2) Entering postgraduate training in a research group with a high scientific reputation and in-depth coaching; those who aim to attain a medical specialist title should enter clinical training only after having acquired basic research knowledge and skills. (3) Choosing superiors interested in research and in supporting their junior staff with adequate resources. (4) Looking for a mentor at the very beginning of one's professional career. As already mentioned, and also reported by other authors [10,13,35,36] mentoring proved to be crucial for a successful and satisfying career, especially for female academics.

### Career barriers

Some career barriers are normal obstacles in a professional career. Scientific progress is not a linear process, there are phases of stagnation. After some time however, the problems have to be analyzed as to whether they have their origin in the research project itself or whether working conditions are not adequate, either due to a lack of support from superiors or a lack of resources. Junior staff have to be courageous to ask superiors for advice and better working conditions, but if nothing improves should look for another post where they get better career support and better research conditions.

An important question brought up by the interviewees has been to what extent it is possible to combine a good clinical career with first class research. In Switzerland, for an academic career in clinical medicine it is still stipulated to be an excellent clinician and a brilliant researcher who bridges basic and applied sciences. A "bedside researcher" or a clinician-educator [21] are not acknowledged as excellence in academia. Therefore adequate structures and resources have to be provided so that young physicians can conduct research projects during regular working hours (i.e. as protected research time) as well as pursue their clinical training. Thus they are not forced to sacrifice their spare time for research. Furthermore, to meet the requirements of high standard research it is necessary to establish research cooperation between clinicians and scientists of different fields. Melhado [37] already pointed out that clinical research has fundamentally changed in the last decades, it needs extensive inputs from various research fields. To establish such a multidisciplinary network junior academics need the support from their senior staff, at least in the beginning of their career.

### Career paths in academic medicine

Physicians come to a career decision at different stages of their postgraduate training. The career plan is often not stable in the beginning. Some physicians who start their postgraduate training in research followed by clinical training will not pursue an academic career, others who experience both good clinical training and good support in their research activities will come to the decision to pursue an academic career in the course of their further training. Structural working conditions and support by superiors play an important role to motivate young physicians for an academic career. The interviewees in this study have come to their decision during six years of postgraduate training. At this stage the aspired to career plan seems to be stable, especially because they have already invested a lot of time, money, and professional commitment.

Four types of career path could be identified in this study. The first type is a career path where physicians mainly *work in basic research*, not followed by clinical training. This is a challenging career, junior scientists can fully concentrate on their research activities; furthermore they usually attend special postgraduate programs and are members of a research group from which they get support. The advantages of this career path are predictable working conditions. This allows for good compatibility of work and family life, which should basically attract more female physicians. In our study none of the female academics pursued this career path. As experienced in medical school classes, those females who choose to study medicine and not biomedical sciences primarily want to work with patients. As a consequence, a career in basic research is not an option for them.

Potential disadvantages of a career in basic research can be that junior academics are more dependent on one single superior, i.e. the group leader in a laboratory. Any disagreement between junior and superior can have a negative impact on the junior's career promotion. Furthermore, physicians who choose this career do not have the option of opening a private practice. Self-employment is hardly possible.

*The second type of career path starts in research, often in a PhD program, before entering clinical training*. It is especially chosen by physicians who want to carry out basic research in a clinical field. This career type meets the current trend, as molecular medicine has also gained significance in clinical medicine. Junior academics trained in basic research and pursuing a clinical career have good promotion prospects. Again, none of the females aspired to this career path. Reasons might be that women want to complete specialty training as soon as possible to have more flexibility later on, if they want children.

The problem with this career type is the physicians' double professional identity: they are basic scientists as well as clinicians; in their work they have to cope with two different methodological approaches: basic research and clinical work. Physicians who pursue this kind of career have to invest in a long postgraduate training period. Later on they are faced with a constantly high workload which poses the risk of a work-life imbalance. This career path contradicts the general trend towards lifestyle preferences reflected in the aspiration for a better work-life balance.

In the *third type of career path *the graduates also start in research. *They usually work just for a year or two in a research laboratory before they enter clinical training*. In this research year they mainly learn research methodology but do not have enough time to gain the skills and knowledge to start their own projects or apply for research grants. They therefore often experience difficulties in continuing their scientific studies along beside their clinical work. Furthermore they lack a research group and a senior researcher to guide them in establishing their own projects. Their main identity is that of a clinician. Those physicians who start to pursue this career path and are not well supported run the risk of dropping out of the academic pipeline.

Physicians who follow the *fourth type *try to *combine their training in research and in a medical specialty in parallel*. This is still the traditional career path. Research issues are derived from clinical work and are investigated in clinical research projects. This career path is near to clinical work which better fits female physicians' interests. In the last few years clinically based research has gained more recognition. However, young physicians interested in clinical work and clinical research often have difficulties with time management and in getting adequate research support and resources. They are at high risk of overcommitment and of developing symptoms of burnout. Because of the high workload they may tend to neglect private life and family.

Considering the aforementioned four career types in medicine it can be stated that a career in basic research does not create conflict between clinical work and research. There is a better compatibility of work and family obligations. For those physicians pursuing an academic career in clinical medicine it seems opportune to follow the second career path with sufficient knowledge and skills in basic research followed by clinical training. However, it should first be mentioned that entering clinical training after three years of research activities in a PhD program is difficult. Second, this type of career path is not suitable for physicians following a career in a surgical field. For them it is advisable to acquire surgical skills as early as possible in their postgraduate training. They should therefore preferably follow career path three or four and depend on adequate support from their superiors with respect to clinical training as well as research activities.

Summarizing it can be stated that the workplace conditions for an academic career in Switzerland are far from being friendly for the young physician generation, especially for the females. The requirements for a specialty qualification in a clinical field as well as those for an academic career with the 'habilitation' as the first step on the hierarchical ladder have been continuously tightened in the last ten years. These measures have mainly been set up by male professors and act as exclusion mechanism for women [38]. Although there is a certain awareness of gender equality, professional opportunity structures and workplace conditions are still not adjusted to women academics in medicine: (1) there exist not enough child care facilities; (2) there are age limits for scholarships, grants and the 'habilitation', which are far-reaching obstacles for female academics with children. Only female professors could force the change, but there are not enough in the decision-making bodies. Some of the older female professors have adapted to the existing career demands, have sacrificed their personal life, and are not willing to fight for their young 'sisters'. Health politicians started to improve the employed physicians' obligatory weekly workload and set a limit of 50 hours. However, in many institutions, headed by male professors, these guidelines are not followed. Another factor which acts as a deterrent to pursue an academic career is the lower remuneration compared to that of clinicians.

Some *limitations *of the present study must be considered: as in most qualitative studies it presents a wide range of differentiated statements from a small number of study participants which is why no generalization can be made in relation to a greater number of academics. Furthermore, the statements refer mainly to postgraduate training in Switzerland. However, a *strength *of the study is that the qualitative data could be complemented by quantitative data. As shown, the interviewees' statements reflect quantitatively gained results.

## Conclusion

The conditions for an academic career in medicine in Switzerland appear to be difficult – especially for those physicians combining research with clinical work. Research activities are not an integrated component of specialist training and time spent on research activities can only be partially accredited for the acquisition of a specialist title. The main recommendation for a successful academic career in medicine is to start early with research activities, for instance to complete preferably two years of research after graduation. When applying for clinical training the junior physician should consider whether the head of the clinical department is interested in research and whether he/she is willing to guarantee time and resources for research. Furthermore, mentoring programs should be established in which junior academics get career advice at an early stage of their career. As seen in a mentoring program at the medical school of Zurich University careful matching between junior academics and mentors who work in similar research fields but are not the mentees' superiors helps reduce the obstacles to junior career advancement and contributes to more goal-oriented planning of subsequent career steps [15]. University mentoring programs can help to overcome gender-related career barriers [13,35,36]. In addition, it is crucial that senior staff believe in the academic power of female physicians, even if they have a family [34]. Last, decision-makers – superiors and health politicians – should take the lead to improve structures and conditions for an academic career in medicine allowing for a better work-life balance.

## Competing interests

The authors declare that they have no competing interests.

## Authors' contributions

BBF is principal investigator, BBF, MS, and CB designed the study. MS conducted the interviews and performed the statistical analyses of the quantitative data. BBF and MS conducted the content analysis. All three authors interpreted the data, BBF drafted the manuscript, critically revised by MS and CB. All authors read and approved the final manuscript.

## Pre-publication history

The pre-publication history for this paper can be accessed here:


